# Attachment classification, psychophysiology and frontal EEG asymmetry across the lifespan: a review

**DOI:** 10.3389/fnhum.2015.00079

**Published:** 2015-02-19

**Authors:** Manuela Gander, Anna Buchheim

**Affiliations:** Institute of Psychology, University of InnsbruckInnsbruck, Austria

**Keywords:** psychophysiology, heart rate, cortisol reactivity, skin conductance, EEG asymmetry, prefrontal cortex

## Abstract

In recent years research on physiological response and frontal electroencephalographic (EEG) asymmetry in different patterns of infant and adult attachment has increased. We review research findings regarding associations between attachment classifications and frontal EEG asymmetry, the autonomic nervous system (ANS) and the hypothalamic-pituitary-adrenocortical axis (HPA). Studies indicate that insecure attachment is related to a heightened adrenocortical activity, heart rate and skin conductance in response to stress, which is consistent with the hypothesis that attachment insecurity leads to impaired emotion regulation. Research on frontal EEG asymmetry also shows a clear difference in the emotional arousal between the attachment groups evidenced by specific frontal asymmetry changes. Furthermore, we discuss neurophysiological evidence of attachment organization and present up-to-date findings of EEG-research with adults. Based on the overall patterns of results presented in this article we identify some major areas of interest and directions for future research.

## Attachment and psychophysiology: an introduction into the field

According to Bowlby (1969) attachment is a biobehavioral state in which several physiological and behavioral systems are organized in order to provide an individual with a certain sense of security and intimacy with significant others. As attachment behaviors seem to be approach and withdrawal behaviors, Bowlby assumed that attachment behaviors are associated with approach and withdrawal mechanisms. For example, children around 6–9 months withdraw from the negative stimulus and approach their caregivers for security (Marvin and Britner, 1999). Bowlby defined these behavioral systems as biological systems that work along with physiological processes. It is assumed that individuals correct and adjust their approach and withdrawal behaviors by learning to manage their environments and maintain the homeostatic balance within the physiological system when they are emotionally aroused (Bowlby, 1969; Bretherton, 1995; Solomon and George, 1999). Mental representations of early attachment relationships shape emotional and cognitive information, which affects our attention and memory as well as our emotional reactivity of the central nervous system. In order to maintain organization within the attachment system, emotional reactivity is then regulated within the central nervous system (Main et al., 1985; Bretherton, 1993). Attachment is associated with different ways to regulate emotions and thus some researchers have actually argued that the attachment system is in itself an emotion regulation device (Vrtička and Vuilleumier, 2012). It has been hypothesized that attachment relationships (at least those with secure attachment classification) have a regulatory effect on individuals’ affective and physiological responses to distress (Diamond, 2001; Gross, 2007).

Research employing physiological measures and electro-encephalographic (EEG) techniques in children, adolescents and adults has flourished in recent years due to increased interest in understanding psychological states and behavior associated with attachment. The psychophysiological approach focuses on the measurement of physiological correlates of observed behavioral responses and the way in which individual differences in physiological response predispose specific behavior. In the present review we aim to present research findings regarding associations between attachment classifications and frontal EEG asymmetry, the autonomic nervous system (ANS) and the hypothalamic-pituitary-adrenocortical axis (HPA) across the lifespan. We included studies that report on participants’ reactions to scenarios involving behaviors that are relevant for attachment purposes like sequences of separation and reunion. Furthermore we discuss reactions as a function of an individual’s attachment classification. As our review is limited to prefrontal activation using EEG, we excluded studies presenting functional neuroimaging data (for further details on fMRI data, see Vrtička and Vuilleumier, 2012).

Before launching into specific areas of neurophysiological attachment research, it is necessary to point out that there are different ways of measuring attachment. In infants, the most commonly used is the Strange Situation procedure, where infants are classified into secure, insecure-avoidant, insecure-ambivalent and disorganized attachment groups. Securely attached infants have confidence in their caregiver’s availability and they actively seek them for comfort and use them as a safe haven. Insecure-avoidant infants expect rejection from their caregiver and consequently avoid their caregiver to effectively reduce anticipated conflict or rejection. Insecure-ambivalent infants are uncertain about their caregiver’s response so they demonstrate passive or angry resistant behavior that serves to establish proximity to their caregiver. Disorganized infants have been unpredictably frightened by their caregiver. They are caught in a conflict when confronted with a situation that normally elicits attachment behavior. Many of them show signs of fear, freezing or disorientation during the Strange Situation procedure (Kobak and Madsen, 2008).

In adults there are two major approaches, namely self-report measurements and narrative methods. Although they both measure and classify attachment groups, they define the construct in a different way. Self-report questionnaires either assess categories of attachment style or they measure the degree to which dimensions of attachment styles are present (Ravitz et al., 2010). The outcome of self-report items is a product of thoughts about attachment that have entered the consciousness of a person and therefore reflect how the individual wishes to represent him- or herself towards others. Several self-report measurements like for example the Adult Attachment Questionnaire and the Experiences in Close Relationships converge on two dimensions of insecurity. Attachment anxiety refers to individuals with a negative sense of the self. They tend to expect separation, abandonment or insufficient love and they are preoccupied with the availability and responsiveness of others. Furthermore, they tend to maximize negative experiences, they are hypervigilant to potential threat and they demonstrate a hyperactivation of attachment behavior. In contrast, attachment avoidance refers to individuals with a negative sense of others. They are characterized by self-reliance, an avoidance of intimate relationships and they devaluate the importance of close relationships. In addition, they minimize feelings of distress and deactivate attachment behavior. Some instruments like for example the Relationship questionnaire allow deriving categories from these dimensional scales. According the Bartholomew and Horowitz’s four category model these categories correspond to combinations of extreme positions on the aforementioned two dimensions. A secure attachment style is defined as a relative absence of attachment avoidance and attachment anxiety. A preoccupied/ambivalent attachment style is characterized by high attachment anxiety and low attachment avoidance. Individuals with an avoidant attachment style have low attachment anxiety and high attachment avoidance. Finally the fearful attachment style is conceptualized as high insecurity on both attachment avoidance and anxiety (Bartholomew and Horowitz, 1991).

In contrast to self-report measures narrative instruments like the Adult Attachment Interview (AAI) and the Adult Attachment Projective Picture System (AAP) do not rely on conscious self-evaluation and they allow analyzing unconscious defensive processes. The AAI is a semi-structured interview that measures the representation of attachment experiences in the mind of individuals. Individuals are asked to retrieve attachment-related autobiographical memories from early childhood and evaluate these memories from their current perspective (Hesse, 2008). According to the AAI there are four main adult attachment representations: Secure-autonomous (F), insecure-dismissing (Ds), insecure-preoccupied (E) and unresolved (U). Secure individuals (F) are able to reflect and integrate positive and negative experiences with their caregivers and their evaluation of attachment experiences is coherent. They have a so called “internalized secure base” which can be interpreted as the ability to internally and mentally explore thoughts and feelings. In contrast to them, insecure-dismissing individuals (Ds) tend to idealize or devaluate their attachment experiences by deactivation of attachment distress. Insecure-preoccupied (E) individuals are enmeshed with their caregivers and they show anger and low autonomy in their narrative evaluation. Finally, the unresolved category (U) refers to individuals who show a breakdown of defensive and coping strategies when talking about traumatic experiences like loss and abuse. Their evaluation is incoherent and often includes fearful affect (Buchheim and George, 2011, 2012). The AAP (George and West, 2012) is an established and validated interview to assess attachment representations, based on a set of eight picture stimuli. The stimuli are line drawings of a neutral scene and seven attachment scenes (e.g., illness, separation, solitude, death, and threat). The AAP classification system designates the four main adult attachment representations identified using the AAI classification system (secure, dismissing, preoccupied, unresolved). The AAP narratives of secure individuals (F) demonstrate the ability and willingness to think about attachment distress. In their stories characters reach out to attachment figures for comfort, they show a lot of constructive actions and they often describe mutual enjoyment in their relationships to others. The insecure-dismissing (Ds) group is characterized by a predominance of deactivating defensive processes that emphasize distance in relationships. Their narratives often focus on achievement and exploration. Attachment relationships usually provide functional care or they are described as authoritarian. The AAP stories of insecure-preoccupied (E) individuals include a lot of material that confuses and obscure attachment relationships. They typically concentrate on emotions related to problems, their responses have several undecided themes or story endings and they often focus on the past rather than on the present. This confusion in the story line is also reflected in the blurring of the hypothetical story with personal experiences. The unresolved attachment (U) refers to a group of individuals who are not able to contain and reorganize stories including features that evidence danger, helplessness, failed protection or isolation. Unresolved individuals become momentarily flooded by their attachment fears that cannot be reorganized in the narratives (George and West, 2012). In sum, attachment style as measured by self-report questionnaires is a product of thoughts that enter an individual’s consciousness whereas developmental attachment patterns assessed by narrative techniques are based on the evaluation of defensive processes. A large body of research confirms a low correlation between self-report and interview results suggesting that they both assess different facets of attachment (Ravitz et al., 2010). Therefore, we can assume that despite they are theoretically related, attachment style and developmental attachment patterns are not the same constructs.

In the field of neurophysiological attachment research, there are studies measuring autonomic activity, which provides an index of physiological arousal like increased heart rate, skin conductance (electrodermal activity) or respiration. The ANS can be divided into the parasympathetic and sympathetic branches, which characterize different stress responses. The parasympathetic branch regulates organ functions. It is responsible for maintaining normal growth, restoration of internal organs, rest-and-digest and feed-and-breed situations. The sympathetic branch is the dominant branch in stress situations and stimulates acceleration in heart rate, increased sweating and blood pressure. In times of intense stress individuals redistribute metabolic resources to deal with external threat. On a physiological level this leads to an activation of the sympathetic nervous system accompanied by some degree of deactivation of the parasympathetic nervous system. As attachment relationships have a regulatory effect on the physiological and psychological response to stress, it is expected that the presence of attachment figures lead to an attenuated activity of the sympathetic nervous system (Diamond, 2001). Other studies measure the activity of the HPA like cortisol change during tasks designed to provoke stress (Phillips et al., 2006; Fox and Hane, 2008). According to Hennessy (1997) hormones that are released by stress-induced HPA activity do not only support the formation of social bonds but they are also influenced by proximity to and separation from social partners. Thus it is important to consider the relational context of support provision and an individual’s interpretation of relational context in order to interpret reactivity to different types of tasks which are performed in social contexts. In order to accurately capture an individual’s HPA response, it is necessary to measure cortisol in salvia or plasma 15–30 min after the completion of the procedure as the cortisol response takes place over a much longer time course than other physiological systems. For analyzing cortisol recovery after a stressful task, samples are usually taken 60 min after the procedure (Laurent and Powers, 2007). From an attachment perspective, it can be assumed that a secure attachment buffers physiological reactivity in response to stressors as these individuals can balance exploration and attachment, they are more open in their emotional expressions and they can use attachment figures as a safe haven. In contrast, insecure attachment is related to deficits in emotion regulation as they either deactivate attachment distress (in case of the avoidant/dismissing group) or they show low autonomy and feelings of anger (ambivalent/preoccupied group). Thus it is expected that these individuals show a heightened physiological reactivity in response to attachment-related stressors. The disorganized/unresolved group demonstrate a breakdown of defensive and coping strategies, suggesting that they might display the highest physiological arousal.

In addition to psychophysiological attachment research, a growing body of work is examining frontal EEG asymmetry and its relation to attachment. Research in that field indicates that there is a relation between approach/withdrawal tendencies and hemispheric asymmetries in EEG activity at frontal electrode sites. Left and right cerebral hemispheres are also involved in the experience and/or expression of different emotions (Fox and Davidson, 1984; Fox, 1991). The main focus of attachment studies is on the hemispheric asymmetry in EEG power in the general range of 3–12 Hz over the frontal region. A particular emphasis is on the “alpha” band (6–9 Hz). Activity in this band has been seen as being inversely related to cortical activity over a given scalp region. The model of Fox and Davidson (1984) proposes that the right frontal activation might be associated with withdrawal behaviors and the expression of negative affect and the left frontal activation might be associated with approach behaviors and the expression of positive affect. A decrement in left frontal activation indicates an absence of the expression of positive affect. A decrement in the right frontal activation it is associated with an absence of the expression of negative affect. In the study of Davidson and Fox (1982) the brain activity of 10 month old infants was recorded while watching videotape segments of an actress who was spontaneously generating a happy or a sad facial expression. In two studies the infants displayed a greater activation of the left frontal than the right frontal area in response to the happy segments as evidenced by lower alpha power on the right side. Other studies found the same asymmetry in frontal EEG activity when infants respond to sweet and sour tastes (Davidson and Fox, 1989) and when mothers or a stranger approach the infant (Fox and Davidson, 1984). Pizzagalli et al. (2005) also argue that resting left prefrontal dominance in the EEG activity might be associated with the propensity of developing approach-related tendencies. These study results suggest that there is an association between the regulation of approach and withdrawal mechanisms within the prefrontal cortex and attachment dependent emotion regulation. In line with the aforementioned hypothesis on physiological correlates, we expect clear differences in the emotional arousal between the attachment groups evidenced by specific frontal asymmetry changes.

## Psychophysiology and attachment classifications

Various data from different studies suggest that there is an association between the regulation of approach and withdrawal mechanisms within the prefrontal cortex, physiological arousal and emotion regulation that is related to attachment issues (Fox et al., 1992; George et al., 1999; Dawson et al., 2001; Roisman et al., 2004; Schieche and Spangler, 2005; Zelenko et al., 2005; Feldman et al., 2011). In this review we report on participants’ reaction to scenarios involving behaviors that are relevant for attachment purposes like sequences of separation and reunion. Furthermore we discuss reactions as a function of an individual’s attachment classification. In the following paragraphs we summarize studies on cardiovascular and galvanic skin response, adrenocortical activity and EEG asymmetry for the different attachment classifications across the life span.

### Cardiovascular and galvanic skin response

Infants gain security from being physically close to a caring caregiver. When growing up children are expected to move from this behavioral level to an internal mental representation of the attachment figure (Main et al., 1985; Marvin and Britner, 1999). It is assumed that the more adaptive strategy of securely attached individuals leads to a lower physiological arousal in heart rate and skin conductance. This might contribute to their ability to deal with negative information in emotional processing (Dozier and Kobak, 1992; Spangler and Grossmann, 1993; Stanley, 2006). In infants, Sroufe and Waters (1977) investigated heart rate changes in the Strange Situation and found that although all attachment groups showed increased heart rate upon separation secure infants managed to recover their heart rate after reunion within less than 1 min after contact with their mothers and heart rate deceleration when they returned to play. In accordance to these results, Donovan and Leavitt (1985) also found acceleratory trends of the heart rate in secure infants. Furthermore, they found out that secure infants displayed heart rate deceleration when the unfamiliar adult approached. This might indicate their ability to direct their attention and orient to a stranger. Interestingly, the heart rate of mothers and secure infants paralleled each other, which might be caused by the mother’s involvement in her infant’s behavior. Zelenko et al. (2005) also found that mothers showed acceleration during separation and deceleration upon reunion that paralleled with that of their secure infants. In contrast to other attachment groups, mothers of securely attached infants displayed a heart rate decrease after they successfully calmed their infants. In a recent study, Feldman et al. (2011) also found a concordance between maternal and infant heart rate. Especially during episodes of affect and vocal synchrony infant and maternal heart rate increased similarly compared to non-synchronous moments.

When a secure strategy fails to gain support and comfort from an attachment figure, individuals develop secondary strategies to regulate the attachment system. Insecurely avoidant/dismissing individuals use deactivating strategies which are characterized by a suppression of emotions and masking of negative affect during attachment related situations. Conversely, insecurely ambivalent/preoccupied individuals are enmeshed with their caregivers and they commonly use hyperactivating strategies indicating that they show more negative affect and less autonomy in attachment related situations (Dozier and Kobak, 1992; Roisman, 2007).

Looking at innovative studies on heart rate and skin conductance in infants, researchers have argued that insecure infants are highly aroused during separation and reunion (Sroufe and Waters, 1977; Dozier and Kobak, 1992; Roisman et al., 2004; Diamond et al., 2006). Sroufe and Waters (1977) reported about the reunion session in the Strange Situation. In contrast to securely attached infants, ambivalent infants were put down before the recovery of their heart rate and so their heart rate was still elevated while playing. Avoidant infants also showed an increased heart rate from beginning of separation long into the reunion. Avoidant infants, on the other hand, minimize the display of negative emotions and thus might be protected against elevated cardiovascular reactivity in response to stressful situations. A recent study by Hill-Soderlund et al. (2008) examined the role of physiology in avoidant infants in comparison to secure infants in the Strange Situation. As the authors expected infants with an avoidant pattern of attachment exhibited a greater vagal withdrawal which indicates greater allostatic load. Surprisingly, they could not find a greater vagal withdrawal during the reunion episode in avoidant infants, although they are expected to be more actively engaged in minimizing their distress. Thus, the primary caregiver probably provides some sort of regulatory repair (Hofer, 2006).

From studies with disorganized infants during the Strange Situation we know that they suffer from tremendous stress as indicated by their increased heart rate and skin conductance when they are alone in the room and in the presence of a caregiver. Spangler and Grossmann (1993) analyzed the cardiovascular response of six disorganized infants. Their results indicate that disorganized infants have a significantly higher acceleration in their heart rate when they are alone in the room compared to the other infants. In accordance to these results, Willemsen-Swinkels et al. (2000) found an association between a disorganized pattern of attachment and an increase in heart rate during parting with the caregiver and a decrease in heart rate during reunion.

A number of researchers have examined the physiological response of adults during the AAI (George et al., 1996). Adults with the classification secure give open, coherent and consistent accounts of their childhood memories, regardless of whether they were positive or negative. They are able to integrate their various experiences into a unitary whole and to reflect upon their accounts during their interviews. These individuals have free access to the topics asked about and show a feeling for balance. It is suggested that a “secure” discourse can be understood in terms of a capacity for fluidly shifting attention between memories and maintenance of coherent discourse with the interviewer. Study results indicate that individuals with a secure attachment representation show a lower increase in skin conductance levels from baseline to questions asking them to recall experiences of separation, rejection and threat compared to the other groups (Dozier and Kobak, 1992, 1993; Roisman et al., 2004).

Dozier and Kobak (1992) found that adults with deactivating strategies (i.e., individuals who often report very positive relationships with their parents, play down the significance of their early attachment experiences and show lacks of concrete episodes) show a marked increase in skin conductance levels and heart rate from baseline to questions asking them to recall experiences of rejection, threat and separation from their parents. Individuals with hyperactivating strategies (i.e., individuals who show negative involvement and excessive detail when reporting relationships with their parents which is generally associated with the preoccupied group) also display higher skin conductance and heart rate when questioned about separation or threatened separation from caregivers (Dozier and Kobak, 1993; Roisman, 2007; Holland and Roisman, 2010).

Beijersbergen et al. (2008) did one of the few studies that investigated physiological reactivity in adolescents during the AAI and a family interaction task. Dismissing adolescents did not seem to be more stressed than secure adolescents in the AAI as indicated by their cardiovascular response. The authors assume that they are probably less open to the challenge of the AAI and can cope with the interview in a somewhat superficial manner. However, they exhibited an increased heart rate and electrodermal activity during the direct interaction task with their mother when trying to find a consensus in an area of disagreement indicating that it seems impossible for them to be uninvolved in direct interaction.

Differences in adult regulation patterns can not only be observed in the AAI but also in other situations where individuals activate their attachment system (Feeney and Cassidy, 2003). For example, Roisman (2007) examined psychophysiological profiles of secure and insecurely attached individuals (classified by the AAI) during marital interactions. During areas of disagreement with their (pre) marital partners, securely attached adults had the lowest increase in electrodermal reactivity indicating their ability to share their thoughts and opinions with their partners. The insecure-dismissing adults demonstrated heightened electrodermal reactivity while they attempted to resolve the conflict in their relationship. The author linked this physiological response to emotional inhibition which means that they want to avoid their spouses when called to resolve problems. This heightened electrodermal reactivity could also be found for general stressors (Carpenter and Kirkpatrick, 1996; Diamond et al., 2006; Kim, 2006; Kidd et al., 2011; Ablow et al., 2013). Compared to the insecure-dismissing group, Roisman (2007) found that insecure-preoccupied individuals displayed an even higher heart rate while conversing with their partners suggesting an emotional overinvolvement.

Although some studies investigated skin conductance and heart rate during the AAI and conflict interaction tasks, most of them focus on secure and insecure attachment. One of the few studies including the unresolved attachment group found no differences in cardiovascular response and skin conductance level between resolved (*N* = 108) and unresolved (*N* = 23) adolescents during the AAI (Beijersbergen et al., 2008). The authors argue that this might be due to the way they measured physiological reactivity as they only focused on reactivity during the trauma, loss and abuse questions although that may also be discussed in other questions. Furthermore, the breakdown in strategy might be very brief so that the physiological changes may also be more momentary than during the entire response to the questions. Stanley (2006) examined skin conductance in unresolved adults (assessed with the AAP, George et al., 1999; George and West, 2012) while watching separation and reunion scenes. The unresolved group showed significantly higher arousal than the secure and preoccupied groups as measured by skin conductance. Interestingly their level of arousal increased during the reunion, which coincides with reports that with increasing exposure to reunion episodes the unresolved group might become dysregulated (Creasey, 2002). Until now it is still unclear how much intensity would be needed to cause dysregulation.

### Adrenocortical activity

According to Hertsgaard et al. (1995) assessing the cortisol level in attachment research can be very useful as the neuroendocrine system might be stimulated when individuals have inadequate coping behaviors or coping sources are not available. Studies on attachment classification and adrenocortical response mainly focus on stress reactivity after a stressful event. Various studies have examined cortisol levels of infants after Strange Situation procedure. For example, Spangler and Grossmann (1993) collected salvia samples immediately before the Strange Situation procedure as well as 15 and 30 min after the end of the Strange Situation procedure. They found a small decrease in the cortisol response for secure infants 30 min after the end of the Strange Situation procedure. It is assumed that securely attached infants have a more adaptive behavioral strategy in response to stressors and thus they display no or only little adrenocortical activity during the procedure. This finding is consistent among a series of infant studies (Gunnar et al., 1996; Spangler and Schieche, 1998). However, adrenocortical responses among insecurely attached infants during the Strange Situation are inconsistent. Spangler and Grossmann (1993) found an increased cortisol level in both insecure-avoidant and insecure-ambivalent infants, whereas Spangler and Schieche (1998) could only observe an increase in the insecure-ambivalent group. In line with that, also Luijk et al. (2010) found higher cortisol levels after the Strange Situation in ambivalent infants. The authors suggest that the “maximizing” strategies (i.e., they maximize their behavior while they are the same time unable to find a state of homeostasis in interaction with their caregiver) of ambivalent infants, lead to a higher physiological arousal than the “minimizing” strategies of avoidant infants (Cassidy and Berlin, 1994). One possible explanation for the aforementioned inconsistences among infant studies is that behavioral factors might be involved the infant’s cortisol response to stress. A number of studies have explored the relationship between inhibition of exploratory behavior and adrenocortical activation during stressful situations. In sum, the findings suggest that inhibition in exploratory behavior leads to adrenocortical activation in stressful situations only in insecure but not in secure infants (Gunnar et al., 1996; Nachmias et al., 1996; Spangler and Schieche, 1998). These effects during the Strange Situation could be replicated in other stressful situations like baby examination with inoculation (Gunnar et al., 1996) or a confrontation with other arousing stimuli (Nachmias et al., 1996).

Furthermore, it is suggested that adrenocortical activity is also influenced by the quality of the caregiver (Gunnar et al., 1992; Spangler et al., 1994). For example, Schieche and Spangler (2005) explored the influence of maternal behavior, infant-mother attachment and inhibition of exploratory behavior on the cortisol level during a problem-solving task in toddlers. In their study, elevated cortisol was associated with low task orientation and exploration in infants. These characteristics were in turn related to low supportive maternal presence and a reduced quality of maternal assistance during the challenge task. In line with other findings adrenocortical activation was not found in infants with low inhibition in exploratory behavior. Among the highly inhibited infants, those with a secure attachment classification showed the usual circadian pattern, i.e., a decrease in cortisol from task onset to 30 min after the task.

Although many infants develop organized strategies to manage stressful events in the presence of their primary caregiver, some display a breakdown of strategy which is associated with specific parenting behaviors such as maltreatment, abuse or neglect. Adrenocortical response of these disorganized infants has been investigated in only very few studies. In the study of Hertsgaard et al. (1995) adrenocortical activity came out most clearly for the six disorganized infants. They found the highest cortisol concentration in 19 month old toddlers who were classified as having disorganized attachments. Also, Gunnar et al. (1996) observed a significantly higher cortisol level in disorganized infants after the Strange Situation. Bernard and Dozier (2010) were the first to investigate infants’ cortisol changes during the Strange Situation and a comparison laboratory task (i.e., play). Disorganized infants displayed increases in cortisol that were significantly different than changes elicited during the free play task. These differences were not found in the organized attachment groups which support the hypothesis that adequate maternal care buffers an infant’s stress reactivity. Even though these preliminary studies offer exciting data of the effects of maternal care on an infant’s stress response, it will be important for future research to examine what particular aspects of maternal care are associated with adrenocortical response to stress.

To date there has been little research on adult attachment and cortisol response. Usually the adrenocortical activity in adults is assessed in the laboratory, comparing stress reactivity between individuals among the different attachment groups. One of the first studies measuring attachment and cortisol response was done by Powers et al. (2006). The authors assessed attachment in 124 adult dating couples with the Experiences in Close Relationships Questionnaire which represents a continuous measure of attachment anxiety and avoidance. They measured their cortisol response during a relationship conflict task. They collected samples 20, 30, 45 and 60 min after the conflict task to analyze stress recovery of the participants. The authors argue that measuring cortisol level after 60 min is an adequate time for cortisol recovery. Their findings demonstrate that insecure-avoidant attachment style in females predicted greater HPA reactivity during the conflict interaction task with their partners. However, avoidant women could recover very quickly. Interestingly the authors found that in men, in contrast to women, insecure-anxious attachment style correlated with higher stress reactivity and it also took them longer to return to the baseline after the conflict. Laurent and Powers (2007) expanded this research by examining moderating effects within the couples. Their results indicate that an avoidant attachment style in both partners leads to an increase in woman’s cortisol response during the conflict interaction task. The authors argue that the mutual avoidance makes it difficult for the partners to negotiate their conflict and the woman’s sense of responsibility for doing it might generate a stress response even though she appears disengaged.

Another study by Quirin et al. (2008) investigated the cortisol response during an acute stressor consisting of a cognitive task with an unpredictable and uncontrollable noise in 48 healthy women. Results demonstrate that attachment anxiety was associated with heightened cortisol to acute stress. Although some studies were done for stressful events and attachment, only very little is known about regarding the association between attachment and cortisol response over the course of the day. To our knowledge there is only one study by Kidd et al. (2013) examining this relationship and they found that individuals with a preoccupied attachment style who are characterized by high anxiety and low avoidance have the highest cortisol level throughout the day. One explanation for this result might be that hypervigilant strategies lead to anticipatory stress appraisals for upcoming events and an inability to downregulate subjective and physiological response.

All of the aforementioned studies employed self-report questionnaires to assess attachment style. There is only one study by Rifkin-Graboi assessing attachment and HPA activity using the AAI. This study investigated relations between attachment and cortisol levels during daily life and interpersonal laboratory challenges wherein college aged men were asked to respond to hypothetical situations concerning separation, abandonment and loss. Results show that dismissing subjects show comparatively higher cortisol during the challenges than the other attachment groups. Unfortunately, current research still lacks data on unresolved attachment status in adults.

### Frontal EEG asymmetry

In the last two decades researchers have started to recognize the central role of the prefrontal cortex in the regulation and expression of emotion. As mentioned in the introduction, right frontal activation in infants and adults is associated with “withdrawal” emotions like distress and sadness, whereas left frontal activation is related to “approach” emotions like joy and interest (Dawson et al., 2001). It is assumed that securely attached individuals show a flexibility of the prefrontal emotion regulation mechanisms. Studies on insecure attachment show that the activation of the right frontal brain areas, usually seen in the insecure-ambivalent/preoccupied group, is associated with the full expression of distress. The activation of the left hemisphere, displayed by the insecure-avoidant/dismissing group, might signal the inhibition of emotional attachment behavior like crying (Calkins et al., 1996; Rognoni et al., 2008; Behrens et al., 2011).

Several studies examined EEG asymmetry and its relation to attachment in human infants in Ainsworth’s Strange Situation (Calkins and Fox, 1992; Dawson et al., 1992, 2001). Various data from a number of different studies have implicated that securely attached infants are more positive, are less stressed in separation segments and engaged their parents in interaction more often (Davidson and Fox, 1989; Fox et al., 1992; Diener et al., 2002). Spangler and Grossmann (1993) examined behavioral organization in securely and insecurely attached infants using the Strange Situation. Their results indicate that when securely attached infants approached their caregivers for comfort during reunion, mothers managed to calm and soothe their infants with their presence (Braungart and Stifter, 1991). Davidson and Fox (1989) studied EEG asymmetry in 13 month old infants and found out that infants with right frontal activation were more likely to cry at maternal separation than those who displayed left frontal dominance. This finding was replicated in another study by Fox et al. (1992) that recorded EEG during brief maternal separation in two sessions and found again that infants exhibiting right frontal activation were more likely to cry at separation from their mothers. These findings suggest that right frontal activation, commonly seen in ambivalent infants, might be associated with a lower threshold for the expression of negative affect. In contrast, left frontal activation, commonly seen in avoidant infants, might be associated with a higher threshold and a disposition to express more positive emotions.

Other evidence for this model stems from studies on EEG asymmetry in infants of depressed mothers. Dawson and her colleagues (Dawson et al., 1992, 2001; Dawson, 1994) found differences in EEG asymmetry between secure infants of depressed and non-depressed mothers in the Strange Situation experiment. Those who were classified as securely attached to their non-depressed mothers showed significantly greater right frontal activity whereas securely attached infants of depressed mothers showed greater left frontal activity when the mother walked to the door. Ordinarily, sadness and withdrawal (i.e., the activation of the right prefrontal region) would be expected at parental separation. This might lead to the hypothesis that infants of depressed mothers who show left frontal activity (i.e., positive emotion and approach) when their mothers walk to the door, inhibit their negative emotions. Another interesting area of research is the study of the relationship between the level of exploratory behavior, the attachment organization and the EEG asymmetry in infants (Calkins and Fox, 1992; Fox et al., 1994; Calkins et al., 1996). Calkins and Fox (1992) examined the relationship among infant temperament, security of attachment and exploratory behavior at 24 months. In their study, 34 infants were classified as secure, 7 as avoidant and 9 as ambivalent. The authors conceptualized the term behavioral inhibition in exploratory behavior as the infant’s tendency to show negative affect in response to new people, places and events. This tendency is displayed by long latencies to approach the unfamiliar adult, high amounts of time spent in proximity to the mother as well as facial and vocal displays of negative affect. In contrast to that, uninhibited exploratory behavior in infants means that they are quick to explore the novel room, approach the stranger with no apparent distress and spending only little time with their mothers. Results indicate that avoidant infants are likely to be uninhibited in their exploratory behavior whereas ambivalent infants are likely to be inhibited in their exploratory behavior. Securely attached infants tend to display behavior in the mean. In a subsequent study, Calkins et al. (1996) extended these findings by examining the role of behavioral profiles (high/low motor activity and positive/negative affect) and brain electrical activity in 4 month old infants to predict inhibited and uninhibited exploratory behavior at 14 month of age. It was hypothesized that the behavioral profiles of infants are associated with particular profiles of brain electrical activity and response to novelty. Results demonstrate that infants with high frequencies of motor activity and negative affect display greater right frontal activation at 9 months. In the laboratory situation they showed more inhibited exploratory behavior, they spent more time in proximity to the mother and it took them longer to approach the unfamiliar adult. Furthermore their mothers reported that they have more social fear than the low motor and positive affect group. This finding is consistent with previous findings (Davidson and Fox, 1989; Spangler and Grossmann, 1993).

An area of most interest with respect to emotion regulation is EEG asymmetry in disorganized patterns of attachment. It is hypothesized that in contrast to the other attachment groups these infants do not manage to create the best possible proximity to an attachment figure because their parents are extremely insensitive or even frightening. This leads to a temporary breakdown in the infant’s strategy to keep close to the attachment figure. They segregate emotions into a separate system and dysregulate when the attachment system is overstressed (Main and Solomon, 1990). This dysregulation might be observed in the prefrontal mechanisms. For example, Dawson et al. (2001) examined EEG asymmetry in disorganized children during the Strange Situation and found that these children had higher activity in both the left and the right prefrontal cortex. Dawson interpreted these results as the intensity the children were experiencing and also as a reaction of the prefrontal cortex to dysregulation. As the intensity of the attachment increased, children with that attachment representation tend to over react.

A lot of studies in adults focus on EEG asymmetry during emotion perception among individuals with different attachment patterns. There is growing evidence that left frontal activation at rest and in response to emotional stimuli is associated with approach system and positive emotion, whereas right frontal activation is related with the withdrawal system and negative emotions. Regarding this aspect, it is assumed that attachment patterns might have a strong impact on individual affective dimensions and modulate the underlying neural activity (Rognoni et al., 2008). Results from studies on EEG asymmetry and insecurely attached adults indicate that dismissing individuals have higher left than right activity in the prefrontal cortex during a baseline measure (Stanley, 2006). It is suggested that even though they are stressed during a baseline measure they tend to explore and approach so that they can deal with the negative emotionality (Braungart and Stifter, 1991; Spangler and Grossmann, 1993). A growing body of research also supports the presumption that this attachment group processes positive stimuli related to close relationships and intimacy as less arousing and thus activates withdrawal neural circuits. However, when confronted with unpleasant stimuli they either activate approach-related neural circuits (i.e., activation of the left hemisphere) or deactivate withdrawal-related right hemisphere circuits, depending on the attachment activation of the stimuli. On the other hand, preoccupied individuals show the opposite tendency by enhancing withdrawal neural circuits in response to fear and stimulating the approach circuits in response to happiness (Calkins et al., 1996; Schmidt et al., 1999; Buss et al., 2003; Rognoni et al., 2008).

A couple of studies have investigated adult attachment and hemispheric asymmetries during visual stimuli. Rognoni et al. (2008) examined whether adult attachment styles using several self-report measures influence subjective and neurophysiological aspects of emotion. The subjects watched emotional video-clips that induced happiness, fear and sadness with attachment-related content. Results reveal that fearful individuals responded with less arousing subjective experience and right frontal asymmetry to positive stimuli whereas preoccupied individuals showed higher arousal feelings and wider frontal left activation. Interestingly the authors observed opposite patterns in response to fear. This lower level of arousal in fearful adults suggest that they restrictively experience positive affect and this could be related to the lower involvement of the left hemisphere in processing positive information. The enhanced right prefrontal activation shown in the preoccupied group when confronted with the fearful stimuli is in line with previous findings of children who exhibit fearful and shy behavior at rest and during stressful tasks and separation (Calkins et al., 1996; Buss et al., 2003). These results are consistent with other studies that examined how attachment styles modulate brain responses to emotional stimuli. For example, Zilber et al. (2007) examined whether adult attachment styles modulate the processing of emotional stimuli. To assess attachment-related social information processing they used scalp-recorded event-related potentials (ERP) while participants watched unpleasant, pleasant and neutral pictures. The ERP waveforms consist of positive and negative components which allow measuring the processing of emotional stimuli. One of these components is the late positive potential (LPP) which is very strong when individuals watch emotionally arousing pictures. It is assumed that the further from neutral a stimulus is perceived to be, the larger is the resulting LPP amplitude. Their main result was that the difference in LPP amplitudes between negative and neutral pictures was found to be the greatest for individuals scoring high on attachment anxiety. These findings were extended by Chavis and Kisley (2012) who also considered the emotion bias of individuals with different attachment styles. They measured ERP during an affective oddball paradigm in which participants viewed positive, negative and neutral images of people. The authors found that avoidantly-attached adults displayed a bias towards more neural activation in response to negative images suggesting a greater motivational relevance of negative stimuli. From an attachment perspective their favoring of negative over positive social stimuli could be a reason for their interpersonal withdrawal. In contrast to them anxiously-attached adults demonstrated a bias towards neural activation in response to positive images which appears to be related to a greater motivational relevance of positive stimuli. The behavior of striving for interpersonal closeness in anxiously-attached individuals might be reflective of their motivational balance favoring positive social stimuli. Compared to the two insecure attachment groups, the secure groups did not favor either positive or negative categories.

In a very recent study by Escobar et al. (2013) this processing of emotional information was further examined by exploring ERP correlates of facial emotion recognition in adolescents with different attachment styles. The authors were especially interested in the P1 component which can be modulated by stimulus type (e.g., comparing faces to words) and the N170 component which is more strongly triggered with facial stimuli than with object or word stimuli. Results show that the insecurely attached adults exhibited larger P1 for face stimuli and attenuated the N170 component over the right hemisphere. These results could indicate that this group does not differentiate between emotions when looking at facial stimuli. This was also observed in another study by Dan and Raz (2012) in which adults with an insecure attachment style were slower and less accurate at differentiating angry from neutral faces. Furthermore, the larger face-elicited P1 in the insecure group might suggest a general state of higher vigilance and the larger N170 when viewing negative face stimuli might relate to their proneness to a negative bias.

All of the above adult studies employ self-report measures and unfamiliar face stimuli. One of the few studies using narrative technique for assessing attachment representation was done by Behrens et al. (2011). The authors explored whether neurophysiological responses to photographs of the own child differed based on attachment status. EEG response records to a total of 100 images of the participant’s parents and child (25 each of positive, negative, neutral and personal) were analyzed among three mothers with three different attachment patterns (preoccupied, dismissing and secure) classified with the AAI (George et al., 1996). The dismissing mother showed significantly stronger left hemisphere activation across all image types whereas the preoccupied mother displayed significantly stronger right hemisphere activation for all images except the neutral ones during which the activation in both hemispheres did not differ. The mother with the secure pattern of attachment showed greater left hemisphere activation for all but parental personal images during which activation did not differ between the two hemispheres. Since this pilot study includes only three participants, a replication of these findings using a larger sample is necessary to draw further conclusions about EEG response to attachment-related visual stimuli.

A relatively new area of research is the study of how attachment classifications influence behavior and psychophysiology to social vs. nonsocial situations and stimuli (Vrtička and Vuilleumier, 2012). A recent study by Verbeke et al. (2014) measured resting-state cortical brain activity using EEG in 35 participants when they were alone in the room (condition 1) and when they were together (condition 2). Individuals who scored higher on anxious attachment style experienced an enhanced alpha, beta and theta power when they were together with another person during the resting session. Interestingly, these results did not occur in the avoidant group. During the task-free resting state procedure implemented in this study, adults with an anxious attachment style fail to have their need for approval met and consequently they become preoccupied with what other people might think about them. The enhanced alpha power observed in anxiously-attached adults might provide protection for this internal information processing by blocking external interferences of the surrounding sensory input. For a deeper analysis of these differences, future research could add further biomarkers like heart rate or skin conductance.

Studies using narrative techniques to measure prefrontal activation in a standardized social context are a very new field of research. Two recent publications studied neural correlates of attachment representations during a virtual game involving unfamiliar peers. White et al. (2012) measured ERPs during the Cyberball experiment, a ball toss game where participants play with two peers online who first include and later exclude the participant. The results show that an insecure-dismissing attachment is related to negative left frontal slow wave during rejection. This wave form may suggest a bias to expect more extensive or lasting exclusion from the group and thus entail a more negative appraisal of the interaction and less approach motivation. In accordance with other findings, the dismissing subjects also underreported subjective distress on self-report although the demonstrated neural activity that is related to an elevated level of distress (Dozier and Kobak, 1992; Roisman et al., 2004). In a subsequent study, White et al. (2013) analyzed neural responses to the reunion phase in the Cyberball experiment. Compared to secure participants, the dismissing group demonstrated a greater increment in the N2 during reunion with excluders suggesting that their expectations for being rejected are more strongly violated by a re-initiation of fair play that follows the exclusion phase. In other words, these individuals tend to have continued expectations of rejection even though they were re-included by their peers.

## Conclusion and directions for future research

Over the last twenty years the psychobiological research on attachment has increased dramatically. The study of neurobiological underpinnings of attachment and its impact on a range of social and affective behaviors is a relatively new field of research. Vrtička and Vuilleumier (2012) published the first review in the field of neuroscience and attachment focusing mainly on fMRI results. In the present review we intended to make an important addition to that by discussing EEG and psychophysiological data from attachment research. Furthermore we provided an overview on recent studies on cardiovascular reactivity (see Table 1), galvanic skin response (see Table 2), adrenocortical activity (see Table 3) and on EEG asymmetry (see Table 4) among the four attachment classifications across the life span.

**Table 1:** **Studies on cardiovascular response in different attachment groups**.

	Study	N	Participants	Gender	Attachment measures
Infants	Sroufe and Waters (1977)		Individual case studies	Mixed	Strange situation
	Donovan and Leavitt (1985)	29	22 B^a^, 4 A^b^, 3 C^c^	Mixed	Strange situation
	Spangler and Grossmann (1993)	41	18 B, 6 A, 6 D^d^	Mixed	Strange situation
	Willemsen-Swinkels et al. (2000)	82	32 children with pervasive developmental disorder, 22 with developmental language disorder and 28 within the normal range	Mixed	Strange situation
	Izard et al. (1991)	54	40 B, 8 A, 6 C	Mixed	Strange situation
	Fox (1985)	60	43 B, 16 A, 1 C	Mixed	Strange situation
	Spangler et al. (2002)	Series of studies	Mothers and infants	Mixed	Strange situation
	Burgess et al. (2003)	140	140 mothers and infants	Mixed	Strange situation, behavioral inhibition, play with unfamiliar peers
	Tharner et al. (2013)	450	450 mothers and infants	Mixed	Shortened strange situation
	Zelenko et al. (2005)	41	23 B, 6 A, 12 C	Mixed	Strange situation
Children	Stevenson-Hinde and Marshall (1999)	126	38 B, 6 A, 8 C	Mixed	Strange situation
	Stevenson-Hinde and Marshall (1999)	126	38 B, 6 A, 8 C	Mixed	Modified strange situation
	Oosterman and Schuengel (2007a)	50	50 parents and their children	Mixed	Observational inhibition scale, separation and reunion procedure
	Oosterman and Schuengel (2007b)	110	60 foster children and 50 control children with their caregivers	Mixed	Modified strange situation
Parents	Donovan and Leavitt (1978)	22	22 mothers and their infants	Female	Physiological reaction to infant’s signals during the feeding session
	Wiesenfeld et al. (1981)	32	16 mothers and 16 fathers with their infants	Mixed	Audio cry segments of own and unfamiliar infants
	Wiesenfeld and Klorman (1978)	17	17 mothers	Female	Videotaped smile and cry segments of own and unfamiliar infants
	Donovan and Leavitt (1989)	48	48 mothers	Female	Response to infant cries
	Donovan et al. (1990)	66	66 mothers	Female	Response to infant cries
	Feldman et al. (2011)	43	43 mothers and infants	Mixed	Face-to face interactions during free play
	Hill-Soderlund et al. (2008)	132	132 mothers and their infants	Mixed	Strange situation
	Donovan et al. (1997)	38	38 mothers	Female	Response to infant cries
Adults	Spitzer et al. (1992)	131	131 adults	Mixed	Response to stressors in social situations
	Palestrini et al. (2005)	17	17 dogs and their owners	Mixed	Modification of strange situation (strange environment)
	Roisman (2007)	80	40 younger engaged couples, 40 mature married couples	Mixed	AAI^e^; conversation about areas of disagreement with their partners
	Fontana et al. (1999)	60	60 undergraduate women	Female	Stressful tasks with and without social support
	Sbarra and Borelli (2013)	89	89 adults	Mixed	Self-report adult attachment style; divorce-related mental recall task
	Kim (2006)	66	33 college couples	Mixed	7 point Likert scale on attachment styles; distress inducing film clips; discussion scenarios
	Carpenter and Kirkpatrick (1996)	34	34 college women	Female	Questionnaire on attachment style; stressful situation with or without presence of romantic partner
	Ablow et al. (2013)	53	53 women	Female	Infant cry segments, AAI^e^

*^a^B, secure attachment; ^b^A, avoidant insecure attachment; ^c^C, ambivalent attachment; ^d^D, disorganized attachment; ^e^AAI, adult attachment interview*.

**Table 2:** **Studies on electrodermal reactivity in different attachment groups**.

	Study	N	Participants	Gender	Attachment measures
Adolescents	Beijersbergen et al. (2008)	156	156 adolescents	Mixed	AAI^a^; conflict interaction task
Parents	Frodi et al. (1978)	128	64 parents	Mixed	Videotaped crying segments of normal and premature infant
Adults	Boukydis and Burgess (1982)	72	36 couples	Mixed	Audio cry segments of infants
	Dozier and Kobak (1992, 1993)	50	50 students	Mixed	AAI
	Roisman et al. (2004)	60	60 adults	Mixed	AAI
	Diamond et al. (2006)	148	148 adults	Mixed	Confrontation with psychological stressors; discussion of attachment-related issues
	Kiss et al. (2011)	82	82 adults	Mixed	Trust game; ECR^b^ scale
	Davydov et al. (2011)	26	26 female students	Female	Emotional film clips
	Holland and Roisman (2010)	114	115 couples at T1^c^, 57 couples at T2^d^	Mixed	AAI; conflict resolution task; self-reports about relationships
	Rochman et al. (2008)	27	27 university students	Mixed	Self-report on unresolved anger towards an attachment figure; sadness-anger induction
	Groh and Roisman (2009)	60	60 undergraduate students	Mixed	Attachment script assessment; audio cry and laughter segments of infants; emotional experience questionnaire

*^a^AAI, adult attachment interview; ^b^ECR, experiences in close relationships; ^c^T1, time 1 refers to the first assessment; ^d^T2, time 2 refers to the second assessment one year later*.

**Table 3:** **Adrenocortical response in different attachment groups**.

	Study	N	Participants	Gender	Attachment measures
Infants	Gunnar et al. (1989)	66	37 B^a^, 10 A^b^, 16 C^c^, 3 D^d^	Mixed	Strange situation
	Gunnar et al. (1996)	73	73 infants	Mixed	Strange situation; clinic exam-inoculation situation
	Nachmias et al. (1996)	77	48 B, 12 C, 13 A, 4 D	Mixed	Strange situation; parent-reported behavioral inhibition
	Spangler and Grossmann (1993)	41	18 B, 6 A, 6 D	Mixed	Strange situation
	Hertsgaard et al. (1995)	38	17 B, 5 A, 1 C, 11 D	Mixed	Strange situation
	Roque et al. (2012)	51	51 infants	Mixed	Positive and negative emotional situations in natural setting;
	Bernard and Dozier (2010)	32	32 infants	Mixed	Strange situation; play
	Luijk et al. (2010)	369	369 infants	Mixed	CIDIe for depression; strange Situation
	Spangler and Schieche (1998)	106	66 B, 21 A, 15 C,	Mixed	Strange situation; maternal report on behavioral inhibition
Toddlers	Schieche and Spangler (2005)	76	23 B, 19 A, 11 C, 23 D	Mixed	Strange situation; problem-solving task; toddler temperament scale
Adults	Quirin et al. (2008)	48	48 women	Female	Laboratory stress task
	Pruessner et al. (2004)	120	120 college students	Mixed	Mental arithmetic task, parental bonding index (PBI)f
	Powers et al. (2006)	248	124 dating couples	Mixed	Conflict negotiation task; ECRg
	Kirschbaum et al. (1995)	66	66 adults	Mixed	Public speaking task with or without social support
	Kidd et al. (2011)	498	498 adults	Mixed	Relationship questionnaire; behavioral tasks; word/color interference task; mirror tracing task
	Laurent and Powers (2007)	398	199 couples	Mixed	ECR; emotionality, activity and sociability questionnaire for temperament; conflict discussion with partner
	Rifkin-Graboi (2008)	73	73 students	Male	AAIh; daily life and interpersonal laboratory challenges
	Kidd et al. (2013)	1807	1807 adults	Mixed	Relationship questionnaire

*^a^B, secure attachment; ^b^A, avoidant insecure attachment; ^c^C, ambivalent attachment; ^d^D, disorganized attachment; ^e^CIDI, composite international diagnostic interview; ^f^ECR, experiences in close relationships; ^g^AAI, adult attachment interview*.

**Table 4:** **Prefrontal EEG asymmetry in different attachment groups**.

	Study	N	Participants	Gender	Attachment measures
Infants	Davidson and Fox (1989)	13	13 infants	Mixed	Periods of maternal approach and separation
	Fox et al. (1992)	46	(1) 33 infants	Mixed	(1) Separation responses at 12 and 24 months of age
			(2) 13 infants		(2) longitudinal separation distress
	Dawson et al. (1992, 2001)	26	26 mothers (12 with elevated depressive symptoms) and their infants	Mixed	Strange situation
	Calkins and Fox (1992)	52	52 infants	Mixed	Strange situation; assessment of temperament and inhibition
	Calkins et al. (1996)	207	207 mothers and their infants	Mixed	Visual, auditory, olfactory stimuli; infant behavior questionnaire; toddler behavior questionnaire; free play
	Hane and Fox (2006)	185	185 infants (61 control, 67 high degrees of negative reactivity, 57 high degrees of positive reactivity	Mixed	Reactivity paradigm; laboratory temperament assessment battery; early social communication scale; maternal caregiving behavior during routine activities in the home
Children	Davis O’hara (2003)	67	67 children (5–7 years)	Mixed	CBQ^a^; separation anxiety test, three stimulus situations (post-maternal separation, happy/sad and separation/reunion)
Adolescents	White et al. (2012)	23	13 secure, 10 dismissing	Mixed	CAI^b^; cyberball social exclusion task; need threat scale
	White et al. (2013)	23	13 secure, 10 dismissing	Mixed	CAI^b^; cyberball social exclusion task; need threat scale
	Escobar et al. (2013)	40	20 secure, 15 dismissing, 5 preoccupied	Mixed	FFI^c^; modified dual valence task; sociodemographic questionnaire
Adults	Stanley (2006)	124	124 college students	Mixed	AAP*^d^*; video clips of separation and reunion
	Zilber et al. (2007)	44	44 undergraduate students	Mixed	ECR^e^; response to 60 images (pleasant, unpleasant, neutral)
	Rognoni et al. (2008)	39	9 avoidant, 14 free, 9 preoccupied, 7 fearful	Mixed	Film stimuli with emotional content; relationship questionnaire; ECR^e^; adult attachment scale
	Behrens et al. (2011)	3	Case study: 3 mothers and their infants	Mixed	AAI^f^; response to 100 images (positive, negative, neutral, personal)
	Fraedrich et al. (2010)	16	16 mothers	Female	AAP*^d^*; presentation of pictures which show infant faces expressing a positive, negative and neutral emotion
	Chavis and Kisley (2012)	42	14 avoidant, 12 anxious, 15 secure	Mixed	ECR^e^; response to 45 images (positive, negative, neutral)
	Weisman et al. (2012)	65	65 adults: new parents, new lovers and romantically unattached singles	Mixed	Response to images of a familiar/unfamiliar infant face and neutral stimuli
	Dan and Raz (2012)	50	50 undergraduate students	Mixed	ECR^e^; response to images of angry and neutral faces
	Verbeke et al., 2014	35	35 undergraduate students	Female	Attachment styles questionnaire; task-free resting-state under condition A^g^ and condition B^h^

*^a^CBQ, child behavior questionnaire; ^b^CAI, child attachment interview; ^c^FFI, friends and family interview; ^d^AAP, adult attachment projective picture system; ^e^ECR, experiences in close relationships; ^f^AAI, adult attachment interview; ^g^condition A, participants sit isolated in a dimly lit EEG lab; hcondition B, two participants sit together in a dimly lit EEG lab*.

In summary, studies using physiological parameters support the hypothesis that a secure attachment representation buffers physiological reactivity in response to stressors. Securely attached individuals are characterized by open, flexible emotional expressions, they are better able to balance exploration (i.e., autonomy strivings) and attachment (i.e., relatedness) and they show a greater openness to explore their own thoughts and feelings. On a physiological level they respond with less cortisol increase, lower skin conductance and more flexible prefrontal mechanisms during attachment-related stimuli compared to the other attachment groups. However, it is still unknown in how far attachment security and physiological functioning are stable at the different stages of development from childhood to adulthood. Investigating childhood and later adult attachment and analyze their influence on adult psychobiological processes could make a significant contribution towards understanding psychophysiology of human attachment.

In contrast to securely-attached infants and adults, those with an insecure attachment show a heightened physiological reactivity to stress, which is consistent with the hypothesis that attachment insecurity is associated with deficits in emotion regulation. During the Strange Situation, ambivalent infants have higher cortisol level and they tend to show more right prefrontal activity, whereas avoidant infants have increased heart rate from separation long into reunion, an increased cortisol level and they tend to exhibit more left prefrontal activity which is associated with the inhibition of emotional attachment behavior like crying. These findings especially among the avoidant attachment pattern are particularly notable as this group typically shows low levels of subjective distress. This discrepancy between physiological reaction and subjective distress was also observed in adults with an insecure-dismissing classification as indicated by their low level of self-reported stress. Thus a central question for future research is whether there is a failure to experience subjective distress or a failure to report it. Although physiological results would actually support the latter explanation, it will be necessary to closer investigate the mechanisms underlying the lack of correspondence between physiological and subjective responses to stress (Diamond and Fagundes, 2010).

Another important issue regarding the study of neurophysiological correlates is that the majority of neurophysiological studies of adult attachment have used self-report measures (Carpenter and Kirkpatrick, 1996; Pruessner et al., 2004; Kim, 2006; Laurent and Powers, 2007; Rochman et al., 2008; Kiss et al., 2011; Dan and Raz, 2012; Sbarra and Borelli, 2013), whereas only a handful of studies have used a narrative approach towards attachment like the AAI or the AAP (Beijersbergen et al., 2008; Fraedrich et al., 2010; Holland and Roisman, 2010; Behrens et al., 2011). Although both approaches have their roots in a common theoretical tradition, the distinctions between these assessments should be discussed. As already mentioned in the introductory section, self-report measures on attachment are commonly used to assess adult romantic relationships by asking about a person’s feelings and behaviors in the context of close relationships. In contrast to them narrative instruments measure attachment representations and they allow an analysis of unconscious defensive processes—a dimension that is not included in self-report measures. When those two approaches are compared, it becomes apparent that the measures are not interchangeable which raises practical questions why they lead to divergent results and which one can accurately capture the constructs fundamental to Bowlby’s attachment theory. Therefore, Roisman et al. (2007) suggest that due to the small overlap between these two attachment measurement traditions, AAI and self-report literatures should not be cited and discussed as if these measures were interchangeable. An important issue for future research will be a neurophysiological distinctive comparison between infant attachment, adult attachment and caregiving patterns to find out more about the underlying mechanisms at different stages of our lives, which shape our emotional and physiological regulation.

A relatively untapped area of research is that of neurophysiological correlates of unresolved attachment pattern in adults. Although there are a couple of studies on infants, there is a lack of studies for adolescents and adults. From studies with unresolved infants during the Strange Situation we know that they suffer from tremendous stress as indicated by their high cortisol level, their increased heart rate and skin conductance when they are alone in the room. Interestingly they also show a higher activity in both hemispheres during the procedure, which might be an indicator of their segregation. In one of the few studies that investigated physiological response in disorganized adults, Stanley (2006) found a higher arousal as measured by skin conductance while watching video clips on reunion scenes, which might be a signal for their dysregulation. However, Beijersbergen et al. (2008) could not find any significant difference in physiological response during the AAI between the resolved and the unresolved group. Future work should focus on that research topic. At present it is still unclear, how much intensity is needed to cause dysregulation in adults and how they react on a physiological level. Therefore, future research should attempt to identify and connect the moment of breakdown with the recordings of physiological reactivity. Our suggestion would be to use the AAP as stimulus material, since that measure was successfully used in experimental settings (like fMRI) assessing neural correlates of resolved and unresolved attachment in different clinical groups compared to healthy participants (Buchheim et al., 2006a,b, 2008, 2012; Buchheim and George, 2011).

Although psychophysiological attachment research remains an active area, there certainly remain some outstanding questions and limitations concerning the methodology. The first consideration refers to the measurement and interpretation of physiological responses such as the autonomic and HPA axis activity. When measuring adrenocortical activity, researchers must carefully think about the time of the day for their sample collection as there are changes due to circadian rhythms. In addition, some research questions require a recording of physiological data during a baseline level to analyze phasic responses. This can be particularly challenging in experiments including infants and young children as they cannot be instructed to sit quietly for a baseline recording. Therefore, researchers usually record physiological responses when the participants are not responding to a strong stimulus which is not equivalent to a baseline measure in adults. In this respect also underlying physiological mechanisms like for example the degree of vagal control of the heart might have a significant influence on the magnitude of physiological response (Fox and Hane, 2008). The second consideration refers to methodological challenges and constraints involved in the recording of infant EEG. These include environmental challenges like setting up an infant-friendly and interference-free lab but also task-related challenges like designing age-appropriate stimuli and paradigms (Hoehl and Wahl, 2012). Although investigating the correlates of frontal EEG asymmetry among the attachment groups is still an active area of research, overarching developmental model of EEG asymmetry has not been developed yet (Saby and Marshall, 2012). Integrating the previously mentioned findings into such a conceptual model that also considers possible neurophysiological origins of the observed asymmetries and their association to affective processes would be an interesting avenue for future research.

Studying the neurophysiology of human attachment has broadened the understanding of the manner in which attachment represents a buffer or a moderator of initial physiological disposition. This also leads to the assumption that the early caregiving environment has an influence on the physiological processes that underlie individual differences in reactivity. The emerging body of psychobiological research on attachment provides us a promising insight into the interplay of biology and the environment and how they influence the human personality.

## Conflict of interest statement

The authors declare that the research was conducted in the absence of any commercial or financial relationships that could be construed as a potential conflict of interest.

## Abbreviations

ANS: autonomic nervous system
HPA: hypothalamic-pituitary-adrenocortical axis
AAI: adult attachment interview
AAP: adult attachment projective picture system.
